# Mpox (formerly monkeypox) in women: epidemiological features and clinical characteristics of mpox cases in Spain, April to November 2022

**DOI:** 10.2807/1560-7917.ES.2022.27.48.2200867

**Published:** 2022-12-01

**Authors:** Alberto Vallejo-Plaza, Francisco Rodríguez-Cabrera, Victoria Hernando Sebastián, Bernardo R Guzmán Herrador, Patricia Santágueda Balader, Lucía García San Miguel Rodríguez-Alarcón, Asunción Díaz Franco, Ana Garzón Sánchez, María José Sierra Moros, Spanish Monkeypox Response Network, Fernando Simón Soria, Berta Suárez Rodríguez, Spanish Monkeypox Response Network, Elena Vanessa Martínez Sánchez, Marta Ruiz-Algueró, Lorena Simón, María Sastre, Nicola Lorusso, Juan Pablo Alonso Pérez de Ágreda, An Lieve Boone, Mario Margolles Martins, Antonia Garí Bibiloni, Laura García Hernández, Óscar Guillermo Pérez Martín, Jacobo Mendioroz Peña, Ana Martínez Mateo, Francisco Javier Roig Sena, Rosa Carbó Malonda, Raquel Morales Romero, Mª del Carmen Pacheco Martínez, Juan Antonio Linares Dópido, Olaia Pérez Martínez, Alonso Sánchez-Migallón Naranjo, Fernando González Carril, Pello Latasa Zamalloa, Eva Martínez Ochoa, Ana Carmen Ibáñez Pérez

**Affiliations:** 1 Coordinating Centre for Health Alerts and Emergencies (CCAES), Directorate General of Public Health, Ministry of Health, Madrid, Spain; 2 Hospital Universitario Puerta de Hierro, Madrid Health Service, Madrid, Spain; 3 National Centre of Epidemiology, Carlos III Health Institute, Madrid, Spain; 4 CIBER in Infectious Diseases (CIBERINFEC), Madrid, Spain; 5 Hospital General Universitario Gregorio Marañón, Madrid Health Service, Madrid, Spain; 6 Members of the National Monkeypox Response Network are listed under Collaborators; 7 CIBER in Epidemiology and Public Health (CIBERESP)

**Keywords:** Monkeypox, mpox, women, epidemiology

## Abstract

Over 79,000 confirmed cases of mpox were notified worldwide between May and November 2022, most of them in men who have sex with men. Cases in women, for whom mpox might pose different risks, are rare, and Spain has reported more than one third of those in Europe. Using surveillance data, our study found similar time trends, but differences in delay of diagnosis, sexual transmission and signs and symptoms between men and women.

Over 79,000 confirmed cases of mpox (formerly monkeypox) were notified to the World Health Organization (WHO) between May and November 2022. Most of the cases were notified by previously non-endemic countries in Europe and the Americas. Spain has been one of the most affected countries worldwide, with more than 7,393 confirmed cases [1,2]. The General Director of WHO declared mpox a Public Health Emergency of International Concern (PHEIC) [3] on 23 July 2022. Human-to-human transmission in the context of this PHEIC has primarily been related to close and direct physical contact with skin lesions, crusts or certain body fluids of an infected person, especially in the context of sexual relations, but uncertainties about modes of transmission and the potential impact on general population remain [4-6].

Most cases in Spain and other countries have occurred in men who have sex with men (MSM) [6]. However, cases in other population groups, such as women, have also been identified. In this study we characterise the mpox cases among women using national surveillance data [7] and explore differences with cases among men to provide information for a better understanding of the disease’s dynamics.

## Mpox surveillance in Spain and analysis performed in this study

This analysis includes all confirmed cases (detection of the monkeypox virus (MPXV) genome by specific or generic PCR for Orthopoxvirus in a clinical sample) in Spain that have been collected following the detection and control protocols established by the National Surveillance Network that covers all regions of the country [8]. A standardised form was used by local epidemiologists when interviewing the cases and included sociodemographic variables such as sex (dichotomic man/woman), clinical features and risk factors and exposures.

We conducted a descriptive analysis of the variables included in the form. We excluded all cases younger than 16 years, as the circumstances of the disease in children would require different analysis than those in adults. Comparisons of qualitative variables were studied using the chi-squared test, quantitative variables using the Mann–Whitney U test. Multivariate logistic regression was performed to adjust measured odds ratio (OR) by sexual transmission mechanism.

## Mpox cases in Spanish women

From 26 April to 21 November, 158 mpox cases in adult women (≥ 16 years) were reported in Spain among 7,393 total mpox cases (2.1%). Women (age range: 16–76 years) were younger than men, with a median age of 34 years (interquartile range (IQR): 29–44) vs 37 years (IQR: 31–44) in men. Two women were pregnant.

## Evolution of the outbreak and delay in diagnosis

Date of symptom onset of the cases was known for 151 women and 6,940 men. Notified cases showed an increasing trend from April to July (epidemiological weeks 17 to 30), followed by an ongoing decreasing trend (epidemiological weeks 31–46), similar in both women and men (Figure).

**Figure:**
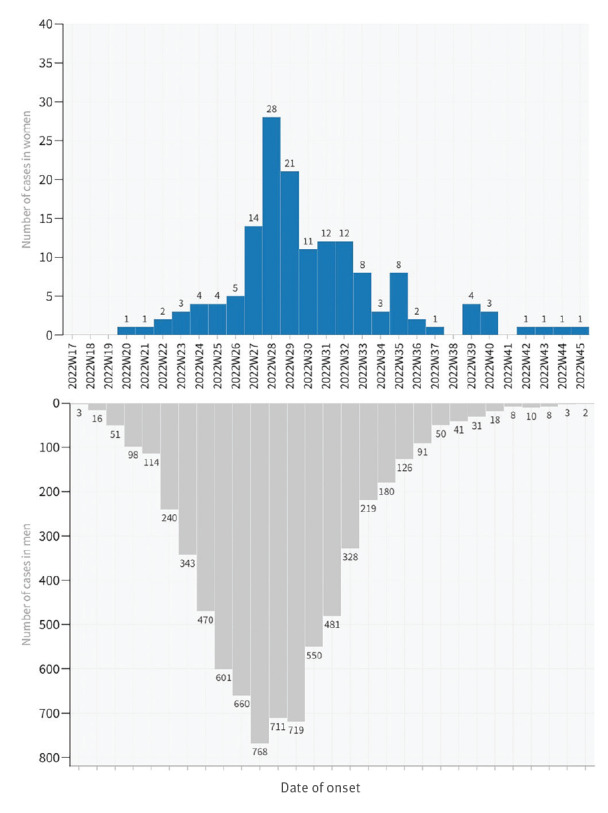
Notified mpox cases with known symptom onset, by sex, Spain, 26 April–21 November 2022 (n = 7,091)

Concerning the delay in diagnosis, we found statistically significant differences (p = 0.042) between sexes. Where information was available (142 women and 6,695 men), the median time from symptom onset to diagnosis was 8 days (IQR: 5–10) in women, compared with 7 days (IQR: 4–10) in men. In hospitalised cases, we found no significant time differences from symptom onset or date of diagnosis to date of admission.

## Sex differences in the mechanism of transmission

The most likely mechanism of transmission was reported for 66.5% of women and 68.0% of men; for both, it was close contact during sexual relationships. However, we found significant differences between men and women with, respectively, 92.9% and 65.7% transmission during close sexual contact (p < 0.001) (Table 1). A study by Thornhill et al., which categorised women as trans, cis and non-binary, also found sexual contact to be the most likely method of transmission for women, in their sample, in slightly higher percentages [9]. 

**Table 1:** Confirmed mpox cases, by sex, Spain, 26 April–21 November 2022 (n = 7,393)

Characteristics	Women (n = 158)			Men (n = 7,235)		p value
	n	%		n	%	
Median age in years (IQR)_)_	34 (29–44)			37 (31–44)		0.023^a^
HIV infection^b^	6		4.4	2,682	40.8	< 0.001^c^
Hospitalised^b^	6		4.1	238	3.7	0.767^c^
**Known transmission mechanism**	**105**		**66.5**	**4,918**	**68.0**	**0.686^b^**
Sexual transmission	69		65.7	4,570	92.9	< 0.001^c^
Human-to-human, non-sexual	23		21.9	324	6.6	
Other, not specified	13		12.4	24	0.5	

IQR: interquartile range.

^a^ Mann–Whitney U test.

^b^ Denominators are the total cases with reported information.

^c^ Chi-squared test.

In addition, 14 women (8.9%) were infected in the context of an outbreak linked to a tattoo studio, included in the transmissions group ‘others’, except for a secondary case, who was human-to-human.

In addition, we found differences in the percentage of cases with HIV infection: 4.4% of affected women compared with 40.8% of affected men (p < 0.001).

## Signs and symptoms in women

The most frequent signs and symptoms reported in women were general symptoms, rash in different locations and local lymphadenopathies (Table 2). The combination of signs and symptoms in women was different than in men: women presented anogenital rash less frequently (51.0% vs 67.3%, p < 0.001) but any rash elsewhere more frequently (71.2% vs 59.1%, p = 0.002). Men and women had oral exanthema and general symptoms in similar percentages (p = 0.535 and p = 0.769, respectively). No differences were found in the number of cases with lymphadenopathies. Differences by sex were still found after adjusting by most probable transmission mode (Table 3). 

**Table 2:** Range of signs and symptoms in mpox cases, by sex, Spain, 26 April–21 November 2022 (n = 7,040)

Signs and symptoms	Women (n = 153)		Men (n = 6,887)		p value^a^
	n	%	n	%	
General symptoms^b^	115	75.2	5,247	76.2	0.769
Anogenital exanthema	78	51.0	4,633	67.3	< 0.001
Oral exanthema	26	17.0	1,307	19.0	0.535
Other exanthemas	109	71.2	4,067	59.1	0.002
Local lymphadenopathies	72	47.1	3,432	49.8	0.497
Generalised lymphadenopathies	6	3.9	368	5.3	0.438

^a^ Chi-squared test.

^b^ Reported any of the following symptoms: asthenia, fever, headache, muscle pain or odynophagia.

**Table 3:** Odds ratio of signs and symptoms in confirmed mpox cases in women compared with men, adjusted by sexual transmission mechanism, Spain, 26 April–21 November 2022 (n = 7,040)

Signs and symptoms	OR (non-adjusted)		OR (adjusted)^a^	
	OR (95% CI)	p value	OR (95% CI)	p value
General symptoms	0.95 (0.65–1.37)	0.770	0.92 (0.72–1.18)	0.721
Anogenital exanthema	0.51 (0.37–0.70)	< 0.001	0.56 (0.45–0.69)	< 0.001
Oral exanthema	0.87 (0.57–1.34)	0.530	0.84 (0.39–0.73)	0.725
Other exanthemas	1.72 (1.21–2.45)	0.002	2.20 (1.74–2.77)	< 0.001
Local lymphadenopathies	0.89 (0.65–1.23)	0.497	0.97 (0.68–1.39)	0.686
Generalised lymphadenopathies	0.72 (0.32–1.65)	0.417	0.46 (0.25–0.83)	0.009

CI: confidence interval; OR: odds ratio.

^a^ Multivariate logistic regression was performed to adjust measured OR by transmission mechanism.

Higher complication rates were reported in women than in men (14.9% vs 8.8%, p = 0.019). When comparing specific complications throughout their clinical process, such as secondary bacterial infections, oral ulcers or keratitis, no statistical significance was found. No deaths have been reported in women, while two deaths were reported among the 7,235 men.

## Discussion

Cases of mpox reported in women in Spain represent more than one third of all cases in women notified in Europe [10]. During the weeks of increasing incidence in Spain (April to July 2022), cases among women were very rare. However, once the disease spread outside the initial clusters and its incidence was close to its maximum, cases among women followed a similar time distribution as cases among men but at a much lower level. As detected in most countries, the main group at risk are MSM who have high-risk sexual relationships [4-6]. Although transmission may happen in other groups, including women, our finding suggests that the monkeypox virus infection has not spread widely to the general population.

One of the main strengths of our study is that we used national surveillance data, including all reported mpox cases in Spain with the early availability of a standardised protocol and an epidemiological form that increased homogeneity of notifications. However, one of the main limitations is the fact that the epidemiological form included, as in all diseases under surveillance in the National Surveillance Network framework, a dichotomous variable for sex, which is a key variable in this analysis. This may have introduced gender misclassification bias in our study. Thornhill et al. classified women as trans, cis and non-binary, finding differences related to gender identity that we could not identify [9], and possibly causing an underestimation of the weight of sexual contact as a method of transmission and the overall risk for women and specifically trans women in our study. In addition, cases were interviewed by many different local epidemiologists, who may have different sensitivity in the information collected for each case. Finally, the number of women among the patients available for analysis was relatively small, although to our knowledge it is the largest available in literature.

Women were diagnosed later than men, which can be explained by several factors. Given the low-risk perception present in the general population, women may have been less likely to associate their symptoms with mpox, thus delaying consulting and testing. Healthcare practitioners may also introduce gender bias in their clinical suspicion of mpox. Previous authors also described symptoms in women, finding comparable results in the frequency of general symptoms, lymphadenopathies and oral lesions, but higher percentage of anogenital exanthemas and any mucosal exanthemas [9].

HIV prevalence rates in mpox cases were significantly higher than the estimated prevalence of HIV in the adult population in Spain (0.1% in women, 0.6% in men) [11]. This suggests that both men and women with MPXV infection are subgroups with characteristics different from the general population.

As for symptoms, we found in the bivariate analysis statistically significant differences between men and women concerning all non-oral rashes, appearing in different locations, but no differences in general signs and symptoms. These differences remain after adjusting for the transmission mode. Keeping in mind the limitations regarding gender classification previously stated, these differences could be explained by several reasons such as a potential difference in exposures and risks concerning sexual practices (e.g. oral, vaginal and anal sex) that may favour transmission, which is an unknown variable that could not be adjusted for.

## Conclusions

To our knowledge, our study describes the largest sample of women with MPXV infection in the available literature. We found that the risk of transmission of mpox in this group exists and must be communicated, although it has remained low throughout the outbreak. Our study provides information relevant to the response to this alert, increasing the knowledge in the disease dynamics in women, and shows how surveillance data can be useful in applied public health research to generate and increase the knowledge about the disease during the outbreak response. Further studies with a more complete classification of sexual orientation and gender identity and the collection of certain risk exposures would help complete the understanding of additional differences in the mechanism of transmission, behaviour and course of the disease in different population groups. 

## Spanish Monkeypox Response Network

Coordinating Centre for Health Alerts and Emergencies (CCAES), Directorate General of Public Health, Ministry of Health: Elena Vanessa Martínez Sánchez. National Centre of Epidemiology, Carlos III Health Institute: Marta Ruiz-Algueró (second affiliation: CIBER in Infectious Diseases, CIBERINFEC), Lorena Simón, Maria Sastre (second affiliation: CIBER Epidemiologia y Salud Pública, CIBERESP). Andalucía: Surveillance and Occupational Health Service. General Directorate of Public Health and Pharmaceutical Regulation, Ministry of Health and Consumer Affairs of Andalusia. Nicola Lorusso. Aragón: Dirección General de Salud Pública. Juan Pablo Alonso Pérez de Ágreda. Asturias: An Lieve Boone, Mario Margolles Martins. Dirección General de Salud Pública. Gobierno de Asturias. Islas Baleares: Servicio de Epidemiología. Dirección General de Salud Pública. Antonia Garí Bibiloni. Canarias: Servicio de Vigilancia Epidemiológica y Prevención, Dirección General de Salud Pública. Servicio Canario de la Salud: Laura García Hernández, Óscar Guillermo Pérez Martín. Cataluña: Servei de Control Epidemiològic i Resposta a Alertes i Emergències. Subdirecció General de Vigilància i Resposta a Emergències de Salut Pública. Jacobo Mendioroz Peña, Ana Martínez Mateo. C. Valenciana: Subdirección General de Epidemiología y Vigilancia de la Salud. Francisco Javier Roig Sena. Rosa Carbó Malonda. Castilla la Mancha: Servicio de Epidemiología. Dirección General de Salud Pública. Raquel Morales Romero. Castilla y León: Servicio de Epidemiología. Dirección General de Salud Pública. Mª del Carmen Pacheco Martínez. Extremadura: Dirección General de Salud Pública. Subdirección Epidemiología. Juan Antonio Linares Dópido. Galicia: Servizo de Epidemioloxía. Dirección Xeral de Saúde Pública, Xunta de Galicia. Olaia Pérez Martínez. Murcia: Servicio de Epidemiología. Consejería de Salud. Alonso Sánchez-Migallón Naranjo. País Vasco: Departamento de Salud del País Vasco. Dirección de Salud Pública y Adicciones. Fernando González Carril, Pello Latasa Zamalloa. La Rioja: Servicio de Epidemiología y Prevención Sanitaria. Dirección General de Salud Pública, Consumo y Cuidados. Eva Martínez Ochoa, Ana Carmen Ibáñez Pérez.

## References

[1] Ministry of Health. Alerta sobre infección de viruela del mono en España y otros países no endémicos. [Situation report on monkeypox virus infection in Spain and other non-endemic countries]. Madrid: Ministerio de sanidad; 2022. Spanish. Available from: https://www.sanidad.gob.es/profesionales/saludPublica/ccayes/alertasActual/alertaMonkeypox/docs/Informe_de_situacion_MPX_20221011.pdf

[2] Centers for Disease Control and Prevention (CDC). 2022 monkeypox outbreak global map. Atlanta: CDC; [Accessed: 20 Oct 2022]. Available from: https://www.cdc.gov/poxvirus/monkeypox/response/2022/world-map.html

[3] World Health Organization (WHO). WHO Director-General’s statement at the press conference following IHR Emergency Committee regarding the multi-country outbreak of monkeypox - 23 July 2022. Geneva: WHO; 2022. Available from: https://www.who.int/director-general/speeches/detail/who-director-general-s-statement-on-the-press-conference-following-IHR-emergency-committee-regarding-the-multi--country-outbreak-of-monkeypox--23-july-2022

[4] European Centre for Disease Prevention and Control (ECDC). Monkeypox multi-country outbreak – first update. Rapid risk assessment. Stockholm: ECDC; 2022. Available from: https://www.ecdc.europa.eu/sites/default/files/documents/Monkeypox-multi-country-outbreak-first-update-8-July-FINAL3.pdf

[5] World Health Organization (WHO). Multi-country monkeypox outbreak: situation update. Geneva: WHO; 2022. Available from: https://www.who.int/emergencies/disease-outbreak-news/item/2022-DON396

[6] Suárez Rodríguez B Guzmán Herrador BR Díaz Franco A Sánchez-Seco Fariñas MP Del Amo Valero J Aginagalde Llorente AH Epidemiologic features and control measures during monkeypox outbreak, Spain, June 2022. Emerg Infect Dis. 2022;28(9):1847-51. 10.3201/eid2809.221051 35820165 PMC9423914

[7] Conde A, Dapena I, Hernández G. Las tecnologías de la información aplicadas a la vigilancia de enfermedades en España. [Information technologies applied to the surveillance of diseases in Spain]. Instituto de Salud Carlos III. [Accessed: 18 Oct 2022]. Spanish. Available from: https://administracionelectronica.gob.es/pae_Home/dam/jcr:35d6af28-9efc-4ae0-81a8-c8ae00cbb52f/48eficiencia.pdfExternal

[8] Ministry of Health and Carlos III Institute. Protocolo para la detección precoz y manejo de casos ante la alerta de viruela de los monos (monkeypox) en España. [Protocol for early detection and case management in the context of the mpox alert in Spain]. Madrid: Ministerio de sanidad/Instituto de Salud Carlos III; 2022. Spanish. Available from: https://www.sanidad.gob.es/profesionales/saludPublica/ccayes/alertasActual/alertaMonkeypox/docs/ProtocoloMPX_20220805.pdf

[9] Thornhill JP Palich R Ghosn J Walmsley S Moschese D Cortes CP Human monkeypox virus infection in women and non-binary individuals during the 2022 outbreaks: a global case series. Lancet. 2022;S0140-6736(22)02187-0. 10.1016/S0140-6736(22)02187-0 36403584 PMC9671743

[10] European Centre for Disease Prevention and Control (ECDC)/World Health Organization Regional Office for Europe (WHO/Europe). Monkeypox, joint epidemiological overview. Stockholm/Geneva: ECDC/WHO/Europe; 2022. Available from: https://cdn.who.int/media/docs/librariesprovider2/monkeypox/monkeypox_euro_ecdc_draft_jointreport_2022-09-21.pdf?sfvrsn=5eceaaad_3&download=true

[11] UNAIDS Data 2019. Global AIDS monitoring 2019: Indicators for monitoring the 2018 United Nations Political Declaration on HIV and AIDS. Geneva: UNAIDS; 2020. Available from: https://www.unaids.org/sites/default/files/media_asset/2019-UNAIDS-data_en.pdf

